# Perioperative management and drug selection for sedated/anesthetized patients undergoing MRI examination: A review

**DOI:** 10.1097/MD.0000000000033592

**Published:** 2023-04-21

**Authors:** Xiaoyu Wang, XueQuan Liu, Junqiao Mi

**Affiliations:** a Department of Anesthesiology, The Affiliated Hospital of Qingdao University, Qingdao, Shandong Province, China; b Julius-Maximilians-Universität of Würzburg, Würzburg, Germany.

**Keywords:** anesthesia management, claustrophobia, deep sedation, drug application, MRI examination, procedural sedation

## Abstract

In recent years, magnetic resonance imaging (MRI) technology has become an indispensable imaging tool owing to significant improvements in MRI that have opened up new diagnostic perspectives. Due to the closed environment, long imaging time, and need to remain still during the examination process, the examiner may cannot cooperate with the completion of the examination of the procedure, which increases the need for deep sedation or anesthesia. Achieving this can sometimes be challenging, especially in the special nontraditional environment of MRI equipment (unfamiliar and narrow spaces, away from patients, strong magnetic fields) and in special populations requiring sedation/anesthesia during examinations, which pose certain challenges for the perioperative anesthesia management of MRI. A simple “checklist” is necessary because it allows the anesthesiologist to become familiar with the particular environment and human and material resources as quickly as possible. For the choice of sedative/anesthetic, the traditional drugs, such as midazolam and ketamine, are still used due to the ease of administration despite their low sedation success rate, prolonged recovery, and significant adverse events. Currently, dexmedetomidine, with respiratory drive preservation, propofol, with high effectiveness and rapid recovery, and sevoflurane, which is mild and nonirritating, are preferred for sedation/anesthesia in children and adults undergoing MRI. Therefore, familiarity with the perioperative management of patient sedation and general anesthesia and drug selection in the MRI environment is critical for successful surgical completion and for the safe and rapid discharge of MRI patients receiving sedation/anesthesia.

## 1. Introduction

Over the past few decades, advances in imaging technology have facilitated advances in the practice of medicine. Especially during the COVID-19 pandemic, medical imaging technology has played an important role in assisting rapid clinical diagnosis and treatment. Compared with other imaging techniques, magnetic resonance imaging (MRI) has the advantage of high resolution of soft tissue and multi-planar reconstruction, which can better display the size, shape, and location of lesions while protecting the patient from the risk of ionizing radiation.^[1]^ More than 80 million MRI exams are performed worldwide each year.^[2]^ It is well-known that there are many unpleasant experiences during MRI scans, such as loud noises, the confined bore of the magnet, and the immobility required to prevent motion artifacts, are the main reasons why patients with claustrophobia, anxiety, or autism are unable to successfully complete MRI exams.^[3]^ Therefore, sedation/anesthesia is recommended for all uncooperative patients (the majority of whom are children) to avoid low-quality imaging due to motion artifacts and thus prolong procedure time. In a 12-year study at 1 university medical center, researchers found an essentially 8.5% increase in anesthesia care during imaging, with a growth rate of 8.1% in pediatric computed tomography and MRI.^[4]^ Achieving this goal is sometimes challenging, especially in the special nontraditional environment of MRI and the particularities of the population undergoing MRI examination, which pose certain challenges to anesthesia management. Until now, almost all publications has focused on studies of sedation for MRI in the pediatric patient populations, and articles on adult patients are scarce.^[5]^ This narrative review aims to summarize perioperative management, special considerations, and drug options in the specific setting of undergoing MRI.

## 2. Reasons for inadequate or incomplete MRI imaging techniques

In daily clinical practice, it is common to encounter situations where an MRI is not completed or cannot provide the required amount of information. Such situations can be classified into: Significant disadvantages of the MRI design include noise and limitations imposed by the narrow bore of the scanner. Noise levels during MRI examinations are usually in the range of 100 to 115 dB, occasionally reaching 130.7 dB, and many MRI scanners are tunnel-like structures with small, dark interior spaces.^[1,6–8]^ Claustrophobia, anxiety, and autism: claustrophobia is a well-known relative contraindication for MRI, and anxiety or fear is triggered by confinement or the prospect of confinement in the MRI environment.^[9]^ Up to 10% of patients cannot undergo MRI imaging due to severe claustrophobia triggered by the small diameter of the MRI tube or loud noises.^[10]^ Moreover, 1 study showed that even in studies where almost all patients completed an MRI exam, up to 37% of patients who underwent an MRI reported moderate-to-high anxiety prior to the scan.^[11]^ In the literature, the rate of failed examinations due to anxiety ranges between 0.7 and 20%.^[3,11,12]^ Autism is a heterogeneous neurodevelopmental disorder that affects the social communication capabilities of a person and the way they perceive their surroundings.^[13]^ The narrow aperture of the MRI scanner, the noise from gradient changes, and the longer unaccompanied time during the scan, combined with the increased rate of anxiety and sensory sensitivity in patients with autism, pose significant challenges for patients undergoing MRI. Involuntary movement during an MRI imaging examination. During MRI imaging, prolonged imaging processes or pain from the disease itself can increase patient discomfort, manifested by increased patient movement and imaging artifacts. The results of 1 study found significant motion artifacts on MRI in 7.5% of outpatients and 29.4% of inpatients and/or emergency patients at a large care center, and 19.8% of all MRI exams had repeated motion artifact sequences.^[14]^ In conclusion, any of the above reasons can lead to MRI imaging results that are of poor diagnostic quality or unsuccessful scans. In order to successfully complete the scan and improve the imaging quality, the need for sedation/anesthesia outside the operating room has increased.

### 2.1. MRI patients with nonoperating room anesthesia (NORA)

The term “nonoperating room anesthesia” (NORA) mainly refers to the anesthesia performed for patients undergoing surgery, diagnostic examinations, or therapeutic operations in places other than the operating room. The following is a procedure for perioperative sedation/anesthesia management in patients undergoing MRI.

### 2.2. Pre-sedation/anesthesia assessment

Significantly, patients undergoing MRI sedation/anesthesia may be more complex than those in the operating room, because pre-anesthesia preparation and patient selection are usually done by others who may not take into account the patient’s physical condition, and the interaction between medications and anesthesics. Anesthesiologists should be aware of any medical conditions or co-morbidities that may increase the likelihood of complications by reviewing the patient’s previous medical records and interviewing the patient or caregiver. These conditions included the patient’s current clinical status, drug allergies, a history of medication use, and a previous history of surgery and implantation materials. A physical examination before sedation should focus on the respiratory and cardiovascular systems. Evaluation of the airways is particularly important, especially in patients with obstructive sleep apnea syndrome and morbid obesity. In addition to routine evaluation, the anesthesiologist must recheck patient implant information to confirm MRI compatibility,^[15]^ here we highlight the importance of MRI information systems that can share patient histories and implants information among the staff involved in MRI imaging. MRI compatibility data for some devices and implants is available online (http://www.mrisafety.com). This is particularly important for new MRIs with higher field strengths, which means there is a higher risk of burns and injuries. Therefore, the anesthesiologists need to formulate anesthesia plans according to the specific situation obtained above, including selecting a reasonable anesthesia method and airway management tools and explaining them to the patient.

### 2.3. Choice of anesthesia method and airway tools

In the background of MRI, the goal of sedation/anesthesia is to facilitate this painless procedure by providing immobility, safety, and comfort; it has been known that adequate sedation significantly improves image quality, thus reducing the need for repeated exams. Four levels in the spectrum or continuum of sedation have been defined by the American Society of Anesthesiologists.^[16]^ These are minimal, moderate, and deep sedation and general anesthesia. Therefore, for the choice of sedation/anesthesia regimen in an MRI suite, it is recommended that mild or moderate sedation be selected in healthy patients who endure the diagnostic procedure. The choice between deep sedation and general anesthesia is based on the specific requirements of the procedure (breath-holding), the child’s complications, and the anesthesiologist’s experience and comfort with deep sedation techniques. MRI is actually a noninvasive method, and there are very few indications for general anesthesia in adults during MRI examinations. Causes include uncontrollable agitation, severe mental retardation, psychological fear, and psychotic syndromes.^[17]^ In contrast, for the pediatric population, the practically of general anesthesia has a place in infant MRI. In children younger than 6 months, strategies whereby natural sleep is induced after feeding and swaddling are generally sufficient to obtain diagnostic images.^[18]^ In children between 1 and 6 years, deep sedation or general anesthesia is often preferred due to lack of cooperation.^[19]^

Regarding the choice of airway management tools, the anesthesiologist should choose a suitable and proficient supraglottic airway (SGA) or endotracheal tube (ETT) based on his own experience and without interfering with imaging. Currently, there are different views on the impact of the different types of SGA on the quality of MRI images. Based on a series of case reports, Kiranpreet Kaur et al^[20]^ believe that i-gel induces the least ferromagnetic interference compared with other SGA devices, improves imaging quality, and generates minimal artifacts during scanning; A previous in vitro simulation study investigated the extent of magnetic susceptibility artifacts caused by 6 different types of SGA devices. That study found a prominent amount of artifacts with the LMA ProSeal, a moderate amount of artifacts with the classic LMA, LMA Unique, and LMA Supreme, and no artifacts with the Ambu Disposable Pharyngeal Mask or iGel.^[21]^ However, F. E. Ucisik-Keser et al^[9]^ believed that no artifacts were found in the above-mentioned LMA laryngeal mask in clinical practice other than the LMA ProSeal, because in all patients the pilot balloons were taped outside the field, as recommended by the instructions.

For patients requiring an endotracheal tube, conventional endotracheal intubation should be used for MRI scans because the reinforcing tube contains metal and is not MRI-compatible, increasing the risk of obstruction. Recently, Japanese researchers have invented a new type of Ti-alloy-reinforced ETT that is less prone to artifacts in high magnetic fields, and this device allows clinical use during MRI.^[22]^

### 2.4. Sedation/anesthesia preparation before MRI scan

Due to the presence of high-intensity magnetic fields in the MRI room, in addition to the patient being compatible with the MRI equipment, in order to prevent the failure of the anesthesia equipment (e.g., display failure and inaccurate measurements) and especially to prevent patient harm, we also need a set of MRI-compatible other anesthesia equipment such as anesthesia machines, monitoring systems, and infusion pumps. Of course, not every facility has MRI-compatible or MRI-safe equipment. In some facilities with limited conditions, it is an option to open a hole in the console wall to penetrate the necessary tubing (drug infusion pump, anesthesia ventilator circuit) from outside the scanner into the patient. On average, each pipeline requires 9.15 meters (30 feet) of pipeline.^[23]^ Anesthesiologists must be familiar with the MRI-specific work environment and available human and material resources prior to sedation/anesthesia and supplement or replace them as necessary. The procedure using a short “checklist” proved useful here because “utility” items such as suction tubes, oropharyngeal airways, laryngeal blades, and handles without them would immediately make the work process difficult, especially in workplaces where they are rarely used (Table 1).

**Table 1:** Brief “checklist” before sedation/anesthesia is carried out.

Checklist	
Overview	1.How much space and time is available to the patient during the scan?
	2.Where is the “CODE BLUE”
	3.Where is the nearest telephone?
	4.Where is the nearest laryngeal mask or tracheal tube?
	5.Where are the safety sockets?
	6.Where is the nearest defibrillator?
Iv. accesses	1.Does the patient have venous access?
	2.Are there extra venous access devices here?
Airway management	Anaesthesia device 1.Are all anesthesia equipment powered on? 2.Have the anesthesia machine and monitor completed the self-check and function normally? 3.Is there enough sevoflurane in the anesthetic vaporizer? 4.Has the soda lime been replaced? 5.Is the suction device connected to the negative pressure system of the examination room? 6.Are the following materials in the anesthesia cart? Tracheal aspirator and oral aspirator, suction catheters; Venturi masks; Adult and pediatric respirationloop Ventilation masks of various sizes, pediatric ventilation masks, endotracheal tubes (cuffed and uncuffed), laryngeal masks; Laryngoscope (handle, blades of various sizes and children’s blades), batteries, shapeable guide wires, stethoscopes, syringes, tapes;
Medicines	Epinephrine, isoproterenol, atropine, ephedrine, amiodarone, dexamethasone, diphenhydramine, methylprednisolone, β-blockers, naloxone, flumazenil, neostigmine
Monitoring	Pulse oximetry (finger and ear clip), Capnography, Blood pressure cuffs of various sizes, ECG cable
Other	Oxygen cylinder, temperature probe

ECG = electrocardiogram.

Induction of anesthesia should be performed in the induction room as the anesthesiologist is not guaranteed free access to the patient’s head in the MRI suite and noise emissions make correct auscultation more difficult. Noise emissions in MRI also require patient hearing protection during examinations. In situations where the patient is sedated, it is advantageous to use a headset with an integrated microphone to enable communication during the MRI. As alarm recognition occurs 34% of the time under ideal conditions.^[24]^ Noisy areas like MRI suites make sound recognition and alarm perception very difficult, we recommend setting the alarm to the maximum level in an MRI environment. Meanwhile, the anesthesiologist should ensure that rescue equipment (such as a defibrillator and difficult airway devices) is functional and readily available prior to the MRI scans.

### 2.5. Considerations during MRI scan

Respiratory depression caused by overdose of sedatives is the most common adverse event during MRI sedation.^[25]^ Hypoxemia should be monitored by pulse oximetry in all patients receiving programmed MRI sedation. Pulse oximetry (SpO_2_) monitoring is a noninvasive method of measuring arterial oxygen saturation and therefore may be delayed when monitoring impaired respiratory activity, especially if the patient is receiving supplemental oxygen. There is evidence that monitoring exhaled carbon dioxide tension is superior to pulse oximetry in patients with early pulmonary hypoventilation. Capnography is the continuous measurement of the partial pressure of carbon dioxide (CO_2_) during inspiration and expiration. The measured partial pressure of CO_2_ at the end of exhalation is defined as the end tidal CO_2_. Information on upper or lower airway obstruction, spontaneous breathing, and circuit leakage can be obtained from the waveform of the end tidal CO_2_.^[16]^ Therefore, capnography is essential for airway management during MRI examinations.

Additional monitoring includes a continuous electrocardiogram. MRI-compatible monitors do not allow ST-segment monitoring because electrical noise generated during MRI scans may distort or completely obscure the electrocardiogram waveform.^[24]^ The only interference-free electrocardiogram available at the moment is provided by Invivo Research Inc., whose product has meanwhile been approved by all MRI manufacturers for triggering the examinations.^[26]^ In addition, noninvasive blood pressure should be measured at least every 5 minutes, and heart rate should be monitored continuously. Circulatory function can be assessed by observing skin color, urine output, and pulse oximetry. Finally, the patient’s body temperature should be measured in case it changes significantly. Patients were transferred to the recovery area after the MRI procedure, and vital signs were monitored at 5-minute intervals until discharge criteria (Aldrete score of 9) were met.^[27]^

Significantly, in the event of an airway emergency or any other hemodynamic emergency during an MRI scan, ferromagnetic equipment (e.g., ambulance, defibrillator, emergency airway equipment) or other personnel must not be allowed into the room, except in most areas where the magnetic field is eliminated. The procedure can take up to 2 minutes to complete, which can be challenging if the patient is hypoxic or hemodynamically unstable.^[5]^

### 2.6. Special considerations for MRI in pediatric populations

Because there are many anatomical, airway, and physiological differences between infants and adults. Anesthesia for pediatric MRIs is particularly challenging. General anesthesia should be preferred in young children due to the inability of pediatric patients to cooperate during prolonged MRI procedures. The clear advantage of general anesthesia for MRI scans is that it is independent of the child’s ability to cooperate. The entire process, including preparation and scan times, is more predictable, and scan quality may benefit because the child is immobilized. Additionally, breath-hold operations can be performed on images that need to be fixed. In this context, anesthesia is an effective quality assurance method. However, because anesthesiologists have limited access to pediatric patients in an MRI setting and because children are less tolerant of hypoxia than adults, the anesthesiologists caring for pediatric patients during MRI scans should be those with pediatric airway and anesthesia management expert. Some scholars believe that the most commonly used airway techniques in children’s MRI studies are the natural airway and the laryngeal mask airway. Ultimately, the choice of airway management depends upon the child’s age, size, and anatomy as well as the anesthesiologist’s preference and the availability of personnel who could help the anesthesiologist if an airway emergency situation develops.

A particular consideration in the pediatric population is the neurotoxicity of anesthetics to the developing brain. In December 2016, the United States food and drug administration issued a warning that reminding children under 3 years of age to repeatedly use prolonged (i.e., more than 3 hours) general anesthesia and sedatives may affect the development of children’s brain.^[28]^ Although neurocognition in the developing brain is challenging to assess given its complexity and the multiple contributing factors involved.^[29]^ A recent multi-national multi-institutional randomized controlled trial with follow-up at 2 years and 5 years has shown that the use of single and short exposures (i.e., <1 hour) of sedation/general anesthesia in this young pediatric cohort is considered safe.^[30–32]^

As a special group of children, the prevalence of autism has increased over the past 5 years. The United States Department of Health and Human Services has recently reported that 1 out of every 54 children aged 8 years old has been identified as autistic.^[32]^ The absence of a clinician and parent during MRI scans under normal circumstances, coupled with the increased rates of anxiety and sensory sensitivity reported in people with autism, can present significant challenges for patients undergoing MRI. Some of these patients can only safely undergo MRI scans under sedation or general anesthesia. Children with autism spectrum disorder (ASD) require special consideration, both in terms of communication and choice of anesthetic technique. There are a few suggestions for the perioperative management of children with ASD. A flexible admission process, minimal preoperative waiting time, and a quiet operating room are ideal.^[33]^ Distraction techniques are recommended to help separate them from their parents, minimize noise and glare, and give them a familiar comfort item in the exam room. In order to ensure the smooth and safe induction of general anesthesia, it is strongly recommended that children with ASD receive premedication. The currently recommended first-line medications are midazolam and dexmedetomidine. This can be especially beneficial for people with severe ASD, who may experience sudden outbursts of emotional aggression toward themselves and others.^[34]^ It is recommended that restraint may be required as a last resort. If restraint is unavoidable, ensure that parents or caregivers understand and agree, and ensure that trained staff are involved. Anesthesiologists should strictly evaluate the interaction of ASD medication and anesthetics before MRI examination. For example, antipsychotics such as risperidone may cause hypotension under general anesthesia and have proarrhythmic properties. They are recommended to be used cautiously. Psychostimulants may increase the sedative dose requirement during anesthesia and may increase the risk of hypertension and arrhythmias, lower the seizure threshold.^[35]^

### 2.7. Special considerations for sedation/anesthetic drug selection in MRI scans

Regarding the choice of sedative/anesthetic agents during MRI examinations, it is clear that a rational choice must be made based on patient compliance, examination time, examination site, and anesthesia method, taking into account the specific characteristics of each patient (e.g., age, previous medication history, co-morbidities, etc). With regard to the choice of sedation or general anesthesia, as previously mentioned, mild or moderate sedation is appropriate for healthy patients with a high degree of cooperation. Deep sedation and general anesthesia are more recommended in patients with severe mental and psychiatric disorders and in children. Most of the current international clinical guidelines on the selection of sedative or anesthetic drugs during MRI have been updated for this special group of children.^[36]^ However, there is no clear indication for the selection of optimal sedative and anesthetic drugs for adults and children. Each clinical center develops its own protocols, including off-label use in some cases because drug licensing varies from country to country. Different pharmacological approaches have specific disadvantages, such as chloral hydrate, which the food and drug administration and European Medicines Agency have withdrawn due to its potential carcinogenic risk, high MRI sedation failure rate, and prolonged awakening. Its approval and use are gradually decreasing, so it will not be described in detail here. A variety of drugs are available for sedation in clinical work. Which of these drugs is beneficial for MRI sedation? Is a single drug better than a combination drug? There is no definite conclusion yet.

## 3. Drugs for sedation or general anesthesia for magnetic resonance imaging

### 3.1. Midazolam

Midazolam is a short-acting benzodiazepine with anxiolytic, sedative, analgesic, and muscle relaxant properties, as well as anterograde amnesia effects.^[37]^ The National Institute for Health and Care Excellence recommends that midazolam be considered one of the first-line sedatives for pain-free imaging procedures because of its broad safety profile. Midazolam is currently available for sedation in a number of ways. Oral midazolam can be used for mild or moderate sedation. Although absorption is very rapid after oral administration, first-pass elimination results in a bioavailability of only 44%.^[38]^ Intramuscular and intravenous administration have a faster onset of action, higher bioavailability, and better controllability. In recent years, there has been an increase in the use of intranasal midazolam for the treatment of claustrophobic MRI patients^[39]^; In a double-blind, placebo-controlled study, Hollenhorst^[40]^ showed that intranasal application of midazolam significantly reduced MRI-related anxiety, resulting in improved MRI image quality. Compared to oral and rectal administration, intranasal administration has the advantage of no first-pass elimination and is effective and well tolerated. It is readily transported through the major paracellular portion of the nasal mucosa, readily crosses the blood-brain barrier, and has rapid and almost complete absorption (approximately 83%).^[41]^ However, some scholars believe that midazolam alone is not suitable for MRI sedation in children because its duration of action is too short to allow a successful 20 to 30-minute procedure. So it must be reinjected or combined with other sedatives such as ketamine and dexmedetomidine.^[42]^ However, it is necessary to pay attention to the risks of respiratory depression when the 2 drugs are combined. Emanuela Inserra et al^[43]^ showed that intranasal dexmedetomidine combined with midazolam provided relatively effective sedation compared with intranasal dexmedetomidine or midazolam alone, with the lowest adverse effects on neonatal MRI scans. Therefore, the combination of dexmedetomidine and midazolam provides a viable alternative for neonates on MRI scans. Small doses of flumazenil are used to reverse midazolam-induced respiratory depression and paradoxical reactions. In short, midazolam is safe as a mild-to-moderate sedative for MRI scans in adults but not as a single sedative for pediatric scans. Meanwhile, we need to pay attention to the risks of a high sedation failure rate, short duration, and respiratory depression with midazolam.

### 3.2. Remimazolam

Remimazolam, an analog of midazolam, is a benzodiazepine and a new ultra-short-acting sedative.^[44]^ Compared with midazolam, remimazolam has the advantages of rapid onset, rapid recovery, and a higher safety profile. Remimazolam can be rapidly hydrolyzed by plasma esterase in vivo, and its metabolites are inactive.^[45]^ In the United States, European Union, and China, remimazolam was initially approved for procedural sedation in adults.^[46,47]^ The current clinically recommended dose of remimazolam for anesthesia induction is 12 mg/kg/hours, the maintenance period is 1 mg/kg/hours, and it can also be adjusted according to clinical parameters (such as heart rate and blood pressure), up to a maximum of 2 mg/kg/hours.^[48]^ Chen et al^[49]^ found that remimazolam had the same sedation success rate as propofol when used for endoscopic sedation, but had a lower incidence of hypotension and hypoxemia, faster recovery time, and no injection pain. Studies have shown that remimazolam (22%) has a lower incidence of intraoperative hypotensive events compared with propofol (49.3%).^[48]^ Unplanned mechanical ventilation is not required during procedural sedation with remimazolam.^[50]^ In terms of recovery after procedural sedation, the mean time from the end of the procedure to being fully awake was shorter with remimazolam [6.0 minutes (95% CI 5.2)] compared with midazolam [12 minutes (95% CI 5.0–15)].^[51]^ The effects of remimazolam can be reversed with fumazenil. Note that there are currently no recommendations for the dose of remimazolam for fumazenil reversal, and it is unclear whether fumazenil reversal of remimazolam would result in subsequent resedation.^[52]^ Moreover, fumazenil is not without side effects, so it is not recommended to routinely reverse the effects of benzodiazepines at this stage. Remimazolam is currently widely used for painless endoscopy (including colonoscopy, gastroscopy, and fiberoptic bronchoscopy) and sedation in ICU patients, and there are no clinical studies on the use of remimazolam for MRI. Combined with its advantages, future trials need to investigate the safety and efficacy of remimazolam for MRI scans.

### 3.3. Dexmedetomidine

Dexmedetomidine is a selective central alpha-2 agonist with sedative, analgesic, anti-sympathetic, and antianxiety properties. The most unique feature of dexmedetomidine is that it preserves spontaneous breathing, which is very important for populations who are prone to respiratory depression, such as those with neurodevelopmental disorders or obstructive sleep apnea, when receiving sedation/anesthesia.^[53,54]^ In addition, the hemodynamic side effects of dexmedetomidine cannot be ignored, such as hypotension and a low heart rate. Because of these side effects, this drug must be closely monitored when used in patients with heart damage. Dexmedetomidine may be administered by oral, buccal, nasal, rectal, subcutaneous, intramuscular, and intravenous (IV) routes. Dosing and bioavailability vary depending on the route of administration. When administered through IV, the average onset of sedation is 8.6 minutes with a recovery time of 41.4 minutes.^[55]^ The rapid phase redistribution half-life is approximately 7 minutes, and the terminal elimination half-life is approximately 2 hours.^[56]^ Its dose for successful sedation is recommended as a loading dose of 1 µg/kg over 10 minutes, followed by a maintenance infusion ranging from 0.2 to 0.7 µg/kg/hours.^[55]^ Buccal (a mean of 2.20 ± 0.38 μg/kg) and intranasal (3 μg/kg) administration of dexmedetomidine may be useful in children with difficult IV cannulation, but their success rates of sedation for MRI are lower than IV administration, and more sedative supplementation is required than IV administration.^[57,58]^ There are several studies on the sedative effects of dexmedetomidine for MRI. Mason et al^[55]^ reported on the MRI scans of 747 children, of which 97.6% were completed successfully. However, 16% of patients experienced cardiovascular side effects (bradycardia never exceeded 20% of the norm). SpO_2_ was always higher than 95% during the examination. In an MRI study of children with obstructive sleep apnea syndrome, a comparison of dexmedetomidine and propofol for MRI sleep induction showed that 88.5% of scans in the dexmedetomidine group were effective sedation that could be provided without the need for additional airway equipment.^[54]^ However, studies have found that with increase in scanning complexity and time, dexmedetomidine alone has a very high sedation failure rate (more than 90%), and its recovery time is about 40 minutes.^[59]^ The desire for more rapid induction of sedation and recovery following sedation/anesthesia has prompted a switch to a drug combination for sedation/anesthesia. In conclusion, due to its unique pharmacological advantages, dexmedetomidine can be used for mild and moderate sedation in MRI examinations. However, due to the high failure rate of a single medication and the long recovery time, it may not be suitable for high-volume medical examination centers. We should explore the best administration method, dosage, and combination selection in the future.

### 3.4. Ketamine

Ketamine is a N-methyl-D-aspartate receptor antagonist with sedative, dissociative, amnesic, and analgesic effects. Administration routes are IV (0.05–2 mg/kg), intramuscular (4–5 mg), oral (5–6 mg/kg), and intranasal (5–10 mg/kg). The onset of action is rapid (1–2 minutes), the duration is brief (10–15 minutes) and the recovery is short (30–60 minutes).^[60]^ The effects of intravenous administration are rapid, and intravenous administration is preferred. Ketamine is attractive because it is a sedative, and respiratory depression is rare, so ketamine is often used for sedation in pediatric populations during MRI. Although ketamine is highly sedative and does not cause respiratory depression, it has a variety of adverse effects, including delirium, agitation, nausea, vomiting, hypersalivation, and laryngospasm, which can be distressing to both the child and the parent. The random movement of the extremities makes the drug less suitable for patients on MRI scans, as it can increase motion artifacts that affect imaging. A study of neurocritically ill patients with brain MRI showed that the efficacy of dexmedetomidine-ketamine sedation was comparable to that of midazolam. Compared with midazolam, dexmedetomidine-ketamine has a shorter scan time and a lower incidence of sedation-related complications (e.g., decreased SpO_2_, hypotension, and aspiration pneumonia).^[61]^ Therefore, ketamine is not recommended as a first-line sedative for MRI, and it is suggested that ketamine can be used in combination with other sedative drugs to reduce the occurrence of its side effects.

### 3.5. Propofol

Propofol is an effective intravenous anesthetic agent without analgesic effects. Propofol has become the first-line sedative for MRI because of its quick action, short recovery time, good antiemetic effect, low incidence of delirium, and high sedation success rate^[62,63]^ Unlike other sedatives, the site of use of propofol is limited to intravenous injections. Note that younger pediatric patients typically require higher doses because of their larger volume of distribution, shorter elimination half-life, and higher plasma clearance.^[64]^ For children undergoing MRI, we recommend continuous infusion of propofol 2 to 5 mg/kg/hours rather than intermittent dosing due to the high magnetic field environment of MRI.^[65]^ The use of an MRI-compatible infusion pump facilitates our continuous use of propofol in specialized settings to achieve adequate and safe sedation in patients undergoing MRI.^[66]^ However, the sedative risks of propofol cannot be ignored either. Propofol usually causes hypotension and dose-dependent respiratory depression, including hypopnea, apnea, and airway obstruction, which may be aggravated in some high-risk populations, such as children, obese patients, and the elderly. In a 7-year study of pediatric MRI sedation/anesthesia anesthesia practice in a tertiary care center, 24,052 MRI scans of patients under 18 years of age were analyzed for anesthetic drug use, trends in use, and related adverse events. The authors noted that although patients in the propofol-dexmedetomidine group had a slightly longer recovery time, the combination of propofol and dexmedetomidine provided a smoother sedation technique, less airway disruption, and a lower dose of propofol. The authors believe that the combined use of dexmedetomidine has a protective effect on neurocognitive adverse reactions. Ultimately, the 2 primary sedation techniques, propofol alone and propofol in combination with dexmedetomidine, have a high level of safety.^[67]^

Therefore, for the choice of propofol as a sedative/anesthetic drug for MRI, the operator should closely monitor the patient’s vital signs and clinical signs, control the airway by placing ETT and LMA, or consider combining drugs to reduce the risk of side effects.

### 3.6. Sevoflurane

Currently, 2 techniques of deep sedation/anesthesia are commonly used in MRI labs for children under 6 years and adults with failed sedation: sevoflurane via LMA or propofol via intravenous access. Both techniques have been shown to be safe and effective.^[67,68]^ However, the administration of propofol at higher doses may increase the risk of airway obstruction and hypoxia, which can be life-threatening if no artificial airway is in place. Moreover, for those who cannot be equipped with MRI-compatible infusion equipment due to limited economic conditions or who do not have intravenous access, continuous infusion of propofol during MRI scanning is even more impossible.

Sevoflurane is the optimal drug for infants and young children, it has shown very good effects in terms of sedation, safety, and manageability and has various advantages such as no airway irritation, stable hemodynamic function, rapid onset, and fast elimination. When undergoing MRI, inhaled sevoflurane has a shorter induction time and faster recovery time than intravenous propofol. However, sevoflurane indicated a greater likelihood of delirium compared to propofol.^[69]^ A retrospective study evaluated the safety and efficacy of MRI scans using a face mask under sevoflurane anesthesia in pediatric patients in a high-volume MRI department. In 7129 patients, the MRI scan time was 17.1 ± 12.4 minutes. The induction and recovery times of sevoflurane were shorter than those of propofol, and the failure rate of propofol was higher. Additionally, sevoflurane is preferred by parents as it is a noninvasive method of administration rather than intravenous.^[70]^ We have known that children affected by neuropsychiatric disorders are considered to be at higher risk of anesthesiologic complications.^[71]^ A university hospital review analysis of the anesthesia records of 1469 children undergoing MRI under general anesthesia over a 10-year period found that sevoflurane-based anesthesia was feasible in 92.3% of cases and the complications rate was low (6.2%; 3.1% respiratory).^[68]^ At present, it is advocated that a single anesthetic is more suitable for anesthesia management in NORA. Interestingly, sevoflurane caters to this feature. Therefore, we strongly recommend sevoflurane as a first-line drug for sedation/anesthesia during MRI scanning.

## 4. Conclusion

Perioperative management of sedation or anesthesia during MRI examinations is the question we help to answer here. Anesthesia management of patients in the special environment of MRI is the challenge for anesthesiologists. Before an MRI scan, a simple “checklist” and understanding of MRI equipment compatibility are necessary, and airway management and resuscitation equipment must be ready and ready to use. Obviously, the choice of anesthesia method and drug must be made on a case-by-case basis, taking into account all the characteristics of each patient. Mild or moderate sedation is suitable for patients with a high degree of cooperation. Midazolam and dexmedetomidine can be the preferred choices, but attention should be paid to the high failure rate of single sedation and its obvious side effects. Combination drugs can be used to balance adverse reactions to other sedatives. Deep sedation or general anesthesia is suitable for children aged 1 to 6 years and people who have failed sedation, and sevoflurane can be used as the preferred anesthetic drug.

## Author contributions

**Conceptualization:** Xiaoyu Wang, XueQuan Liu, Junqiao Mi.

**Supervision:** Junqiao Mi.

**Writing – original draft:** Xiaoyu Wang, XueQuan Liu.

**Writing – review & editing:** Junqiao Mi.
